# Uveitis and nail psoriasis in a patient without articular involvement: a case report

**DOI:** 10.1186/s12886-022-02596-4

**Published:** 2022-09-24

**Authors:** Juan Sebastián Pineda-Sierra, Luisa Fernanda Peña-Pulgar, Carlos Cifuentes-González, William Rojas-Carabali, Alejandra de-la-Torre

**Affiliations:** 1 Neuroscience Research Group (NEUROS), Neurovitae Research Center, Escuela de Medicina Y Ciencias de La Salud, Universidad del Rosario, Carrera 24 # 63C-69, Bogotá, Colombia; 2 Ophthalmology Interest Group, Neuroscience Research Group (NEUROS), Neurovitae Research Center, Escuela de Medicina Y Ciencias de La Salud, Universidad del Rosario, Bogotá, Colombia

**Keywords:** Psoriasis, Nail Psoriasis, Uveitis, Anterior uveitis, Case report

## Abstract

**Background:**

This study aimed to report a case of bilateral anterior non-granulomatous chronic non-infectious uveitis associated with isolated nail psoriasis without articular involvement.

**Case presentation:**

A 55-year-old man with a history of open-angle glaucoma was referred to our uveitis and ocular immunology center with intraocular inflammation concordant with chronic non-infectious bilateral anterior non-granulomatous uveitis. At presentation, he had moderate inflammation in the anterior chamber bilaterally and lesions characteristic of nail psoriasis. Nail psoriasis was later confirmed by nail ultrasonography performed by a radiologist who specialized in psoriasis. Appropriate clinical and paraclinical assessments were conducted, ruling out all other possible causes of uveitis. The patient required dual systemic immunomodulatory therapy with methotrexate and adalimumab, topical anti-inflammatory drugs (steroidal and non-steroidal), and anti-glaucoma therapy to achieve satisfactory inflammatory and ocular pressure control.

**Discussion and conclusions:**

This is the first report of non-infectious uveitis associated with nail compromise in a patient without other manifestations of psoriasis. Despite reports on the relationship between psoriatic disease and uveitis, there is insufficient information on clinical phenotypes associated with uveitis that could lead to later diagnosis and treatment of associated intraocular inflammation. Clinicians should be aware of all subtypes of psoriasis that increases a risk of developing uveitis in these patients.

## Background

Uveitis is an ocular inflammatory disease with various clinical entities and threatens visual function [1]. Non-infectious uveitis is associated with multiple immune-mediated disorders such as psoriasis, inflammatory bowel disease, ankylosing spondylitis, and Behçet’s disease [1]. Most of the current evidence focuses on the association between uveitis and psoriatic arthritis (PsA) [2–4], and a few studies reported cases of uveitis associated with psoriasis without arthritis [5]. Herein, we describe a unique case of HLA-B27-negative non-infectious bilateral chronic anterior uveitis related to isolated nail psoriasis.

## Case description

A 55-year-old man with a history of open-angle glaucoma, urolithiasis, sinusitis, and asthma presented with bilateral conjunctival hyperemia accompanied by pain, photophobia, and decreased visual acuity. He presented for consultation with a diagnosis of idiopathic bilateral uveitis made in another center, where infectious and autoimmune diseases were ruled out through laboratory and imaging investigations (Table 1a).

**Table 1:** Ancillary investigations to determine the etiology of uveitis

Ancillary investigations	Results	Reference values
**a. Ancillary investigations before uveitis consultation**		
PPD	3 mm	0–10 mm
FTA-ABS	Not reactive	
VDRL	Not reactive	Reactive/Not reactive
RF	Negative	0–20 UI/mL
Chest X-ray	Normal	
**b. Ancillary investigations during follow-up**		
Blood count	Normal	
HLA-B27	Negative	Positive/Negative
ANAS-IIF	Negative	Positive/Negative
Anti-CCP	Negative	0–17 U/mL
Hands and axial X-ray	Normal	

*PPD* Purified protein derivative for tuberculosis, *FTA-ABS* Fluorescent treponemal antibody test absorption test, *VDRL* Venereal disease research laboratory, *RF* Rheumatoid factor, *ANAS-IIF* Anti-nuclear antibodies indirect immunofluorescence, *anti-CCP* Anti-cyclic citrullinated peptide

In the first ophthalmological center, he was initially treated with 17.5 mg/week of methotrexate (MTX), which was suspended for 3 weeks due to bacterial sinusitis. Consequently, the uveitis relapsed 3 months later with bilateral grade II pigment dispersion in the anterior chamber (AC), mild inflammation in the AC (0.5 + cells) of the right eye (OD), and moderate inflammation in the AC (1 + cells) of the left eye (OS). Therefore, 20 mg/day of prednisolone and topical prednisolone were added to the treatment regimen alongside 17.5 mg per week of MTX administered orally. Two months later, ocular inflammation worsened, and treatment failure was considered.

**Fig. 1:**
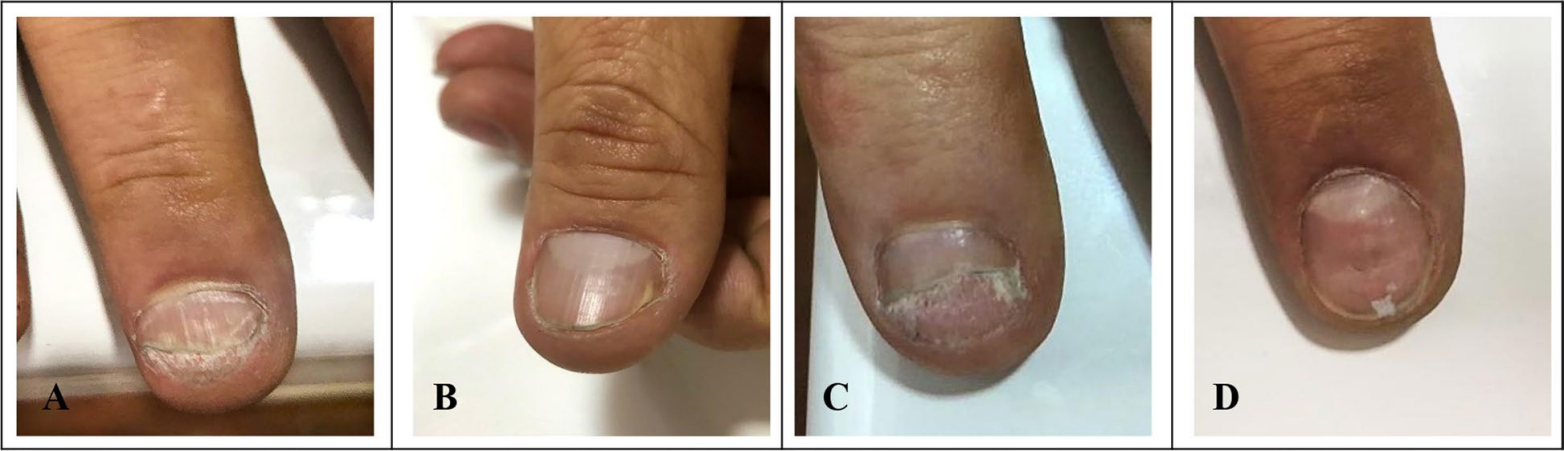
Macroscopic findings on the nails. **a** Third finger of the right hand showing sub-ungual hyperkeratosis, crumbling, leukonychia, distal onycholysis, and pitting. **b** First finger of the right hand showing distal onycholysis. **c** Fifth finger of the right hand with sub-ungual hyperkeratosis, crumbling, and distal onycholysis. **d** Second finger of the left hand showing distal onycholysis and pitting

Subsequently, the patient was referred to our center for a second opinion. During the physical examination, attention was brought to the nails because the patient showed characteristic lesions of nail psoriasis, including pitting, onycholysis, crumbling, and sub-ungual hyperkeratosis along the second, third, and fifth fingers on the right hand and the first finger of the left hand (Fig. 1). Appropriate anamnesis and physical examination were performed to exclude any skin involvement suggestive of psoriasis and any signs or symptoms concordant with PsA.

**Fig. 2:**
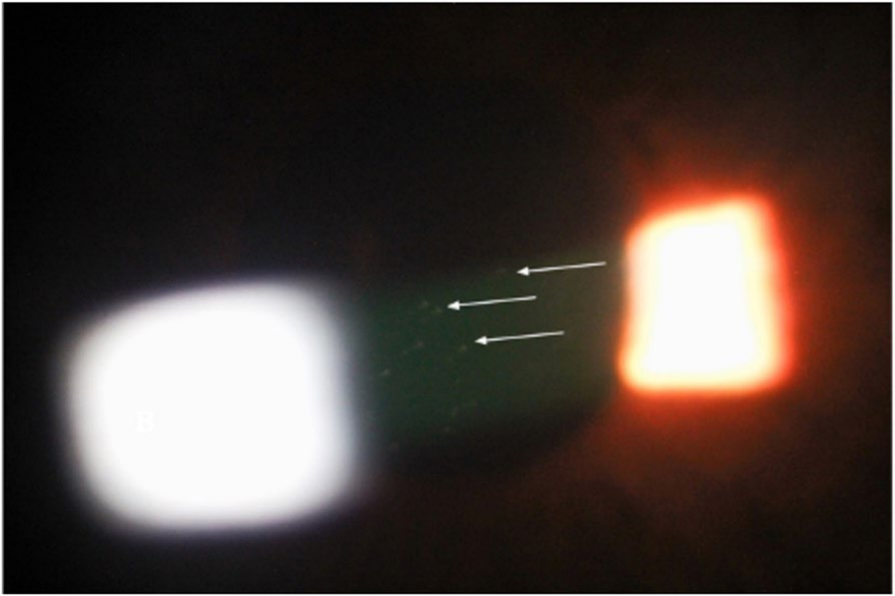
Slit-lamp findings OD. The image shows 1 + cells in the anterior chamber according to SUN grading

On ophthalmological examination, the best-corrected visual acuity was 20/20 in both eyes (OU). Positive findings at slit-lamp examination were Meibomian gland dysfunction, moderate conjunctival hyperemia, gerontoxon, and AC inflammation with 1 + cells according to the SUN grading OD (Fig. 2) similar findings with the only difference being AC cells graded as 0.5 + OS. Nuclear lens opacity was evidenced OU. The intraocular pressure was 20/20 mmHg. A cup-to-disk ratio of 0.9 OU was observed with nasalization of the vessels, and the rest of the posterior segment evaluation was unremarkable.

**Fig. 3:**
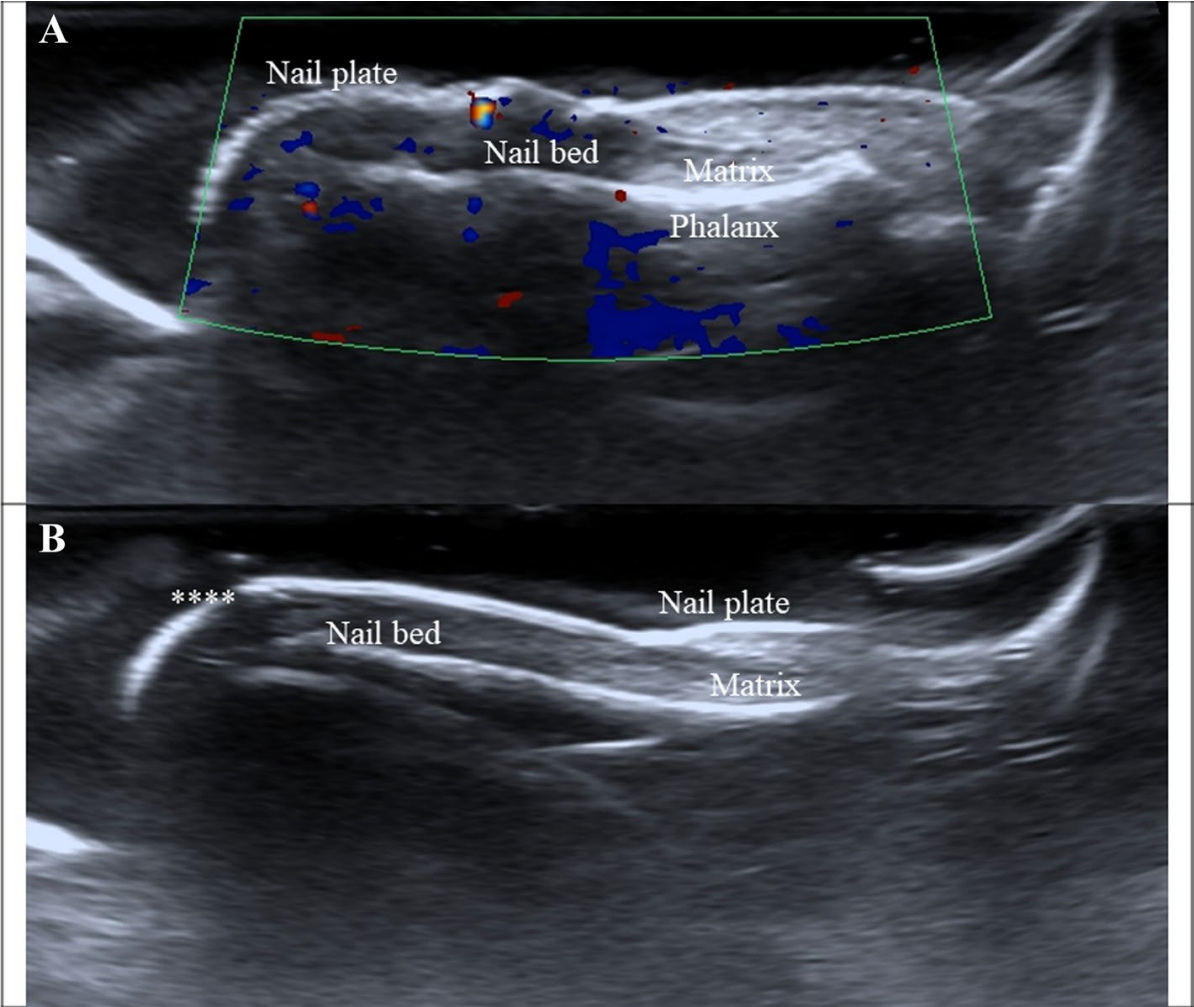
Nail ultrasonography. **a** Grayscale longitudinal plane ultrasonography and Doppler ultrasonography of the nail apparatus of the right hand fifth finger characteristic of phase III psoriatic onychopathy. The image shows wavy appearance with thickening of the nail plates, the nail bed shows focal hyperechoic involvement and thickening. The matrix does not have any changes. The nail vascularization is normal, as shown in the Doppler exploration. **b** Grayscale longitudinal plane ultrasonography and Doppler ultrasonography of the nail apparatus of the left hand fifth finger showing signs of early-stage phase I psoriatic onychopathy. The nail plates have normal morphology with hyperechogenic foci below the distal third of the ventral plate (****). The nail bed, matrix, and distal interphalangeal joint are normal

In this patient with non-granulomatous anterior uveitis with apparent autoimmune/autoinflammatory etiology with nail findings suggestive of psoriasis, PsA had to be excluded alongside any other possible uveitis etiologies not discarded by the previous ophthalmologist. Even though the patient only presented with findings suggestive of nail psoriasis, comparative radiographs of the hands and the axial skeleton did not show positive results. In addition, nail psoriasis was confirmed by nail ultrasonography performed by a radiologist specialized in psoriasis (Fig. 3), and several ancillary tests were performed to rule out other systemic diseases, including anti-nuclear antibodies, anti-cyclic citrullinated peptide, differential blood count, and HLA-B27 typing, and all were normal (Table 1b).

After confirming the diagnosis of HLA-B27-negative anterior non-infectious uveitis, the patient continued the treatment regimen ordered by the previous physician for 6 months before consulting to our center, consisting of 12.5 mg per day of oral prednisolone, topical prednisolone acetate 1%, topical tacrolimus 0.03%, and 17.5 mg per week of oral MTX alongside topical hypotensive treatment for open-angle glaucoma composed of timolol 0.5%, brimonidine tartrate 0.2% and brinzolamide 1%. However, satisfactory inflammation was not achieved 2 weeks after the first consultation in our center showing 1 + cell grading OU. Therefore, dual immunomodulatory therapy with a single dose of 80 mg followed by 40 mg every 15 days of adalimumab was ordered, accompanied by 20 mg per week of subcutaneous MTX, tapering doses of topical and oral prednisone, topical anti-inflammatory therapy with ketorolac tromethamine 0.5%, and topical treatment for glaucoma described previously, achieving overall satisfactory inflammatory response without further relapses (within 6 months). During the 24 months of follow-up, the patient has not referred any PsA symptom.

## Discussion and conclusion

Psoriasis is a common inflammatory skin disease and affects over 60 million people worldwide [6]. It may manifest itself through several clinical phenotypes, some of which are far more common than others, including psoriasis vulgaris or plaque psoriasis, guttate psoriasis, erythrodermic psoriasis, pustular psoriasis, flexural psoriasis or inverse psoriasis, sebopsoriasis or scalp psoriasis, palmoplantar psoriasis, and nail psoriasis [6, 7]. Skin manifestations usually precede nail psoriasis by up to 10 years [8], and it also highly correlated with PsA [8]; however, it can be also present at the onset of psoriasis in 5–10% of patients [9].

The pathophysiological relationship between uveitis and psoriasis has not been fully elucidated; regardless, uveitis has been frequently observed in cases of psoriasis vulgaris and PsA, especially among men [1, 5, 10–13], mainly with HLA-B27 positivity [14]. Tanaka et al. [15] reported an unusual distribution of uveitis in Japanese patients, being most common in psoriasis vulgaris (7/13) than in PsA (4/13). They also reported cases of uveitis associated with pustular psoriasis (2/13) and psoriatic erythroderma (1/13). Nonetheless, previous studies have not described the association between isolated nail psoriasis and uveitis [1, 5, 15].

Uveitis most generally follows the skin manifestations of psoriasis [10, 11] and serves as a possible warning sign of PsA [1]. Still, uveitis can precede skin manifestations of psoriasis in a few cases [10]. Several authors have described the clinical picture of uveitis associated with psoriasis as chronic, bilateral, and severe [1, 10].

Our patient presented a clinical picture of uveitis similar to that reported by Durrani and Foster [5] in patients with psoriasis without arthritis. However, our patient has not yet developed other signs of psoriasis besides the nail lesions. To our knowledge, this is the first reported case with these characteristics. Previous studies have described that PsA can precede the presentation of uveitis by an average of 9.7 (range 0–29) years [16]. In one study of patients with early-stage PsA, 22 (9%) of 242 presented iridocyclitis at the time of diagnosis and 11.3% during follow-up [17]. This study also found that the presence of iridocyclitis upon diagnosis was significantly associated with dactylitis (*p* = 0.032), which was absent in our patient [17]. Therefore, although we cannot discard skin or articular involvement in the future, the time between uveitis and PsA presentation would be unusual.

This case highlights the importance of ophthalmological follow-up in these patients, making it possible to promptly recognize intraocular inflammation in patients with nail psoriasis, even those who have not yet presented with skin or articular manifestations because uveitis may affect the quality of life [18] and highly compromise visual function [19].

In conclusion, we describe a case of bilateral non-granulomatous chronic non-infectious uveitis associated with nail psoriasis. Although uveitis has been associated with psoriatic disease, such an association is limited to a few disease phenotypes. Current published studies on this topic mainly focus on PsA. Therefore, this case should alert ophthalmologists of the possible associations between different disease phenotypes, such as isolated nail psoriasis and uveitis. This could guide recommendations for early ophthalmological examination and uveitis screening to achieve opportune identification, prompt diagnosis, and better visual prognosis for these patients.

## Data Availability

The data analyzed in this study is not publicly available due to protection of medical data privacy but are available on reasonable request from the corresponding author.

## Abbreviations

MTX: Methotrexate
OD: Right eye
OS: Left eye
OU: Both eyes
PsA: Psoriatic Arthritis
